# Food marketing and gender among children and adolescents: a scoping review

**DOI:** 10.1186/s12937-021-00706-4

**Published:** 2021-06-07

**Authors:** Luciana Castronuovo, Leila Guarnieri, María Victoria Tiscornia, Lorena Allemandi

**Affiliations:** 1 Fundación Interamericana del Corazón Argentina, Arévalo 2364 1° A, 1425 Caba, Argentina; 2 PanAmerican Health Organization (consultant), Calle 43 n 1095, 6360 General Pico, La Pampa Argentina

**Keywords:** Food marketing, Gender perspective, Children and adolescents, Scoping review

## Abstract

**Background:**

Pervasive marketing of unhealthy foods is a contributing factor to the growth of the global epidemic of childhood and adolescent overweight and obesity. Sex and gender differences come into play in the design of and responses to these marketing strategies, contributing to the perpetuation of stereotyped behavior and generating disparities in food choices and health. The purpose of this paper is to review the current literature regarding gender differences in food marketing design and perception among children and adolescents to facilitate evidence-based policy dialogues to address gender-based health disparities in NCD prevention.

**Methods:**

Scoping review of articles published in scientific journals in English and Spanish, from 2003 to 2018, that addressed the influence of food marketing among children and adolescents including a gender perspective. The methodological quality of each article was assessed following criteria specific to each study design.

**Results:**

From a total of 37 articles (39 studies) included in the review, 17 were experimental and 22 had descriptive, cross-sectional designs. Twenty-one studies were found to have low methodological quality, while 10 and 8 were of medium and high quality, respectively. A total of 23 studies among children and adolescents found gender-based differences. Differences were found in the following dimensions: food marketing on intake; responses to specific marketing; perceptions and attitudes towards food marketing and marketing regulation initiatives; exposure to food advertising and gendered marketing content. The evidence was not conclusive in any of the dimensions.

**Conclusions:**

The evidence suggests that food marketing has a similar impact on the consumption of unhealthy foods on boys and girls, but boys were found to be exposed to food advertising more intensively and their preferences to be more affected by this exposure, coinciding with a male-dominant advertising content. Limitations of these studies include taking gender as an unproblematic construct equivalent to biological sex and the lack of studies focused on developing countries. As gender is a cross-sectional dimension that interacts with other factors driving health disparities, an integrated gender perspective is needed to develop effective, evidence-based policies to control food marketing and tackle the childhood overweight and obesity pandemic.

## Background

There is no doubt that the current childhood overweight and obesity epidemic is one of the most serious public health issues globally. The most recent global data suggest that 5.6% of all children under the age of five were overweight in 2019, following an upward trend over the past 20 years [1], while in 2016, 18% of the global population 5 to 18 years old had overweight or obesity [2]. Overweight and obesity can cause many physical conditions as well as psychosocial effects, such as negative body image, reduced self-esteem, social discrimination/isolation and, depression [3, 4], thus hindering the comprehensive development of children and adolescents.

The influence of food advertising on eating behaviors, particularly those that target children and promote the consumption of energy dense, processed food products that are high in sugar, salt, and fats, may be contributing to the prevalence of overweight and obesity in children [5–9]. There is a growing body of evidence demonstrating that children’s TV shows in Latin America include more food ads than programs targeting the general public; moreover, the food products marketed to children on TV have a lower nutritional quality than products advertised to other audiences [10]. This has also been documented in Argentina [11, 12]. The food industry invests heavily in marketing these products, most of which do not comply with current dietary guidelines, such as sugar-coated breakfast cereals, sweets and candy, soft drinks, and fast food [13]. They also employ a wide range of media and a diversity of strategies to deliver their messages, including advertising in school environments, food packaging, TV, internet, and social media platforms [10, 14, 15].

International recommendations to tackle the overweight and obesity epidemic [7, 13] include the restriction of food advertising, underscoring the urgent need to implement policies that limit, or even eliminate, the exposure of children and adolescents to food products with low nutritional quality marketing.

Sex (biology) and gender (cultural) differences are relevant to overweight and obesity [16]. Gender differences in food choices and dietary patterns are framed within social factors including societal expectations and stereotypes for males and females being transmitted via parental, peer and media influences. A research conducted by Sweeting [17] showed small differences among male and female obesity rates and no gender predominance within particular age groups. Another study showed that in high income countries, such as Singapore, Denmark and Canada, the prevalence of obesity in boys was almost two-fold greater than girls when comparing within age group [16].

Studies found gender differences in patterning of body fat, the fat levels at which health risks become apparent, levels of resting energy expenditure and energy requirements, ability to engage in certain physical activities, and the consequences of obesity for the female reproductive system. Cultural differences identified in the study include food choices and dietary concerns, overall physical activity levels, body satisfaction and the long-term psychosocial consequences of childhood and adolescent obesity [16].

In this context, it is necessary to gain better understanding of the interplay of gender and food marketing. The evidence shows that advertising tends to offer and exacerbate traditional and stereotyped images of men and women and gender roles. Advertising often contributes to consolidate gender stereotypes that perpetuate culturally rooted social and health disparities and condition differential food choices [18–20].

Here we present a scoping review of literature dealing with food marketing aimed at children and adolescents that also include a gender perspective. This analysis was conducted in the framework of the multi-component regional research project “*Food Marketing targeted to kids: a collaborative and policy-oriented study in Argentina, Bolivia, Guatemala and Peru”*. The overall purpose of this review was to assess the current state of the evidence regarding gender differences in the influence, design, and perception of food marketing directed at children and adolescents and to identify knowledge domains that would benefit from further inquiry. The ultimate goal of this study was to facilitate evidence-based policy dialogues to reduce gender-based health disparities in NCD prevention policies.

## Methods

For this investigation, a specific protocol was elaborated, [21]. We followed the guidelines proposed by Levac, Colquhoun and O’Malley [22] to conduct scoping reviews. We identified and assessed the methodological quality of published scientific articles that addressed the influence of food marketing on eating behaviors among children and adolescents including a gender perspective. Specific themes were gender differences in: a) the effect of food marketing on food intake; b) the effect of food marketing in food choice and preferences; c) responses to specific marketing strategies and techniques; d) perceptions and attitudes towards food marketing and the need for its regulation; and e) advertising content and exposure.

### Inclusion criteria

Studies were considered eligible for this scoping review if they met the following inclusion criteria:

- presented evidence pertaining to children and adolescents up to 18 years old;
- was published in Spanish or English from 2003 to 2018;
- addressed at least one of the following dimensions of interest:
  a theoretical discussion of the association between food marketing strategies and gender disparities;
  a model to estimate how food advertising incorporates gender in combination with other attributes (age, race, socioeconomic level);
  differential food marketing strategies to target specific genders;
  an evaluation of gender-based differences in the influence of food marketing on eating behaviors;

Editorial and commentary pieces, grey literature and articles that did not report specific data pertaining children and adolescents were excluded from this study. Studies including young adults were included if the mean age of the study population was less than 18 years old.

### Search strategy

An electronic database search was conducted using PubMed and EBSCOHost; the latter includes Business Source Premier, CAB Abstracts, EconLit, LISTA, SocINDEX, Cairn.info, Directory of Open Access Journals (DOAJ), ERIC, HeinOnline, JSTOR, OECD iLibrary, Persée, SciELO, World Bank eLibrary, MEDLINE, EMBASE and LILACS. The structure of the search was [(food marketing AND gender) OR (food marketing AND femininity) OR (food marketing AND masculinity)] AND (child OR children OR teenager OR boy OR girl). Additional articles for potential inclusion were identified in a second stage by hand-searching the reference lists in relevant articles.

### Study selection and data extraction

The search was conducted in September and October of the year 2018. Two researchers conducted a two-stage screening process to identify articles that met all inclusion criteria. Two reviewers completed data extraction and another researcher supervised the process.

First, titles and abstracts were analyzed to exclude clearly irrelevant articles and remove duplicates, and then the eligibility of these pre-selected articles was confirmed by evaluating the full text. Disagreements regarding inclusion/exclusion were settled by discussion between the three researchers.

The following information was collected for each included study: full reference (authors, year of publication, journal), country, study design, objectives and main results. The main themes and sub-themes explored in each paper were identified and a narrative synthesis was developed inductively based on these themes.

### Quality assessment

The quality of each article was evaluated using different guidelines that establish the criteria to be met for each study design. Relevance, Appropriateness, Transparency, Soundness (RATS) guidelines [23] were used for qualitative studies, Effective Public Health Practice Project (EPHPP) guidelines for experimental studies [24], and CEBMa for cross-sectional designs [25]. The selected studies were classified according to the percentage of criteria met: low (< 33%), medium (34–66%), and high quality (> 66%).

Studies that analyzed advertising content were evaluated using an instrument specifically designed for this purpose, based on the methodology proposed by Leamy and colleagues [26]. For these studies, a tool was developed, taking into consideration the one described in a systematic revision that took place previously [27]. The dimensions and definitions of the tool were discussed within the research team, and the tool was applied to a subsample. An ordinal scale of three levels/grades was elaborated to evaluate the quality of the different dimensions (1 = the indicator is completely reached; 2 = the indicator is partially reached; 3 = the indicator is not reached). With this scale, the papers can be classified between 5 and 15. This type of scale has been used in previous studies [26] and is adjusted to Cochrane recommendations. The instrument was tested in a pilot study. Agreements and disagreements were discussed among the research team.

## Results

**Fig. 1:**
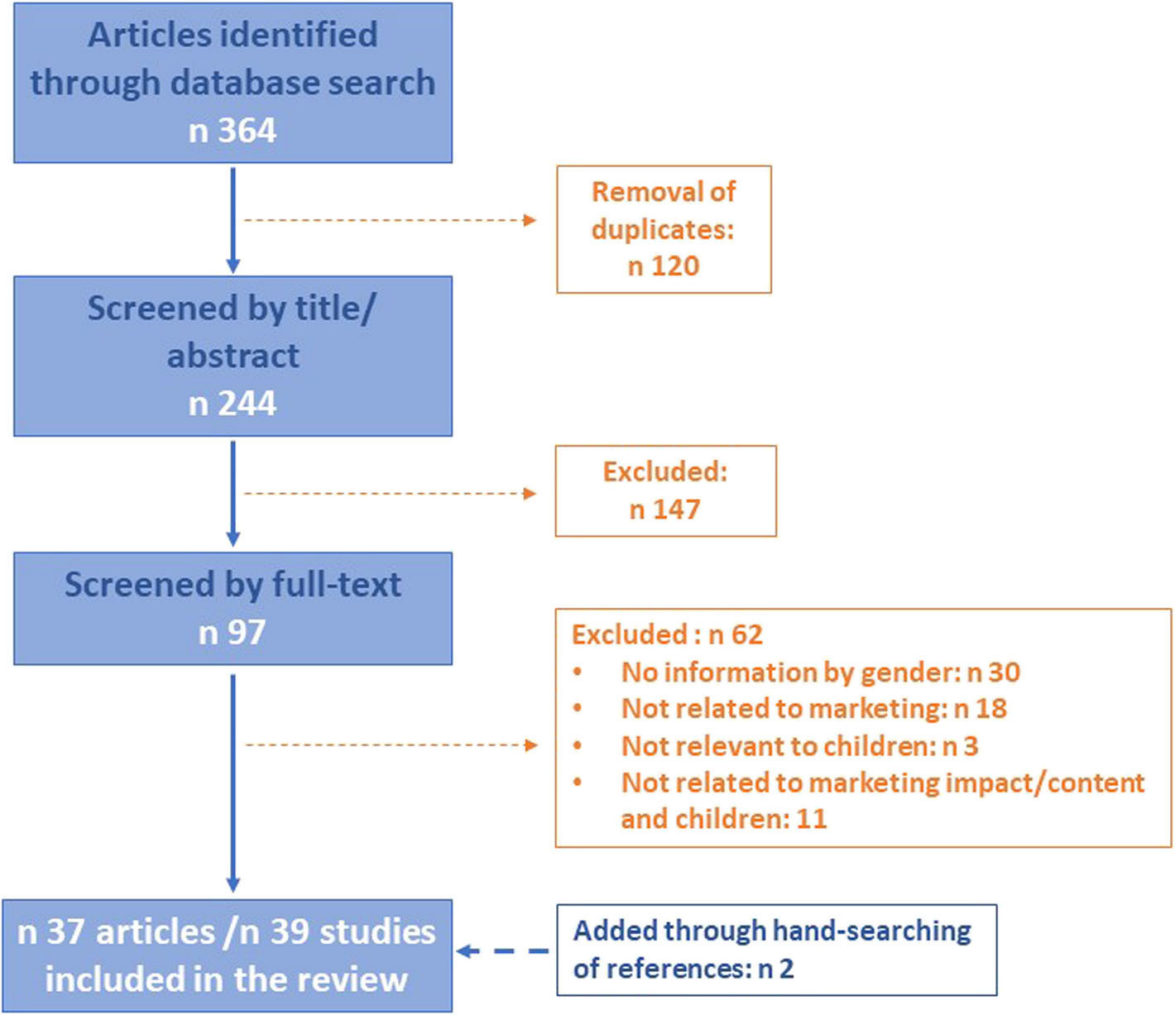
Flow chart of the selection of studies for inclusion in the review

Electronic searches in PubMed and EBSCOHost yielded 364 potentially relevant articles, of which 120 were duplicates and were removed from the list. Screening of title and abstracts excluded 147 articles, leaving a total of 97 potentially relevant articles of which 62 were excluded after full text assessment. The remaining 35 articles were included in this review. By analyzing the reference lists in these articles, an additional 2 were identified as relevant for this work, resulting in 37articles selected for the review (Fig. 1). A total of 39 studies (Table 1) were analyzed -two articles included two separate studies each- of which 17 were experimental and 22 had descriptive, cross-sectional designs. Over half the studies (n 21) were found to have low methodological quality, while 10 and 8 were of medium and high quality, respectively (Table 2).

**Table 1:** Studies included in the review. Summary and main findings

Reference & Country	Study design	Quality score	Population	Objective	Main outcomes
Ueda et al., 2012 [28], India	Experimental	LOW	Children (3–13 years old).	To evaluate exposure to advertising of less healthy food and its association with eating behaviors and BMI	Brand logo recognition: 30 to 80%. Capacity for logo recognition increased with age and socio-economic status. Adjusting by these variables and gender, logo recognition was associated to higher BMI and nutritional knowledge, but not with preferences towards less healthy foods or purchase requests. No significant differences were found between genders.
Chernin, 2008 [29] USA	Experimental	LOW	Children (5–10 years old).	To examine the influence of two advertisements (breakfast cereal, juice powder) on food product preferences	Exposure to food advertising increased children’s preference for the advertised products. This influence was stronger among boys than among girls, although both genders were depicted in the advertisements.
Tarabashkina et al., 2016 [30] Australia	Experimental	MEDIUM	Children and adolescents (7–13 years old).	To assess the role of product evaluations, nutritional knowledge and awareness of persuasive intent on food choices among children and adolescents	When participants showed little nutritional knowledge and low awareness of persuasive intent behind advertising and believed the advertised product to be healthy, they were more likely to choose the advertised product. No significant differences were found by gender and age in the control and experimental groups.
Norman et al., 2018 [31] Australia	Experimental	MEDIUM	Children and adolescents (7–12 years old).	To evaluate the impact of advertising (TV and online gaming platforms) on the amount of food consumed among children and adolescents	Children exposed to food ads in TV and online gaming platforms ate more food while snacking, compared to the group exposed only to non-food ads in TV. There were no significant differences or interactions by age, gender, brand recognition, and household weekly income.
Anderson et al., 2015 [32] Canada	Experimental	LOW	Children and adolescents (9–14 years old)	To evaluate the influence of food ads in TV on the energy intake	Girls with excess weight showed a higher increase of their energy intake compared to girls with normal weight and boys, suggesting higher vulnerability to food advertising. Girls exposed to food ads were more likely to find the TV show acceptable, compared to girls that viewed non-food ads. Among boys, TV show acceptability was not influenced by the product type advertised.
Velazquez and Pasch, 2014 [33] USA	Experimental	LOW	Children and adolescents (8–15 years old).	To assess the relationship between attention to unhealthy food ads and food preferences and choices	The amount of time and frequency of exposure to unhealthy foods was significantly associated to preferences for unhealthy foods. This association was not affected by sex, age or BMI.
Keller et al.,2012 [34] USA	Study 1:	LOW	Children (4–6 years old).	To determine if the presence of a familiar brand affects test-meal intake	Energy intake increased by ~ 41 kcal in children with excess weight when presented with branded food items than when offered unbranded foods (i.e. in unmarked, plain white containers). In contrast, children with normal weight consumed ~ 45 kcal less when presented with branded foods, compared to the unbranded condition. There was no significant difference between boys and girls.
	Experimental				
	Study 2: Experimental	LOW	Children (7–9 years old).	To determine if the presence of a familiar fast-food brand affects test-meal intake	The boys’ energy intake was similar in the presence of fast-food branded/unbranded food items. In turn, girls consumed ~ 100 kcal more when the items were accompanied by a fast-food brand.
Anschutz et al., 2009 [35] Netherlands	Experimental	LOW	Children (8–12 years old)	To evaluate the effects of TV food ads on concurrent non-advertised sweet snack food intake	Boys who viewed food ads presented higher snack intake than boys exposed to neutral (non-food) ads. In turn, snack intake was slightly lower among girls when they viewed food ads than when the viewed neutral ads.
Dixon et al., 2014 [36] Australia	Experimental	LOW	Pre-adolescent children (average: 11 years old).	To evaluate responses to promotional elements -nutrient content claims, sports celebrity endorsements and premium offers- in food packaging	Children were more likely to choose energy-dense, nutrient-poor food products when their packs showed nutrient claims (both genders) or sports celebrities (only boys), compared to control conditions (no promotions).
Hobin et al., 2012 [37] Canada	Experimental	LOW	Children (6–12 years old)	To determine if children make healthier food choices if toy premiums are only offered with healthier fast-food meals	Children were more likely to choose healthier meals when toy premiums were only offered with meals that met nutritional criteria. This effect was stronger among boys than among girls.
Ogle et al., 2017 [38] USA	Experimental	LOW	Children (6–9 years old)	To determine if adding licensed cartoon characters to healthy food/beverages packaging can increase attention to and preference for these products	Children paid more attention to products with characters and preferred less-healthy products, although they preferred products without characters over 60% of the time. Age, sex, and the specific cartoon character were significant influences on product choice, with characters being preferred by younger boys.
Adams and Geuens, 2007 [39] Belgium	Experimental	LOW	Adolescents (15 years old)	To examine responses to healthy and unhealthy slogans in ads for food products perceived as healthy or unhealthy	Although no significant main effects were found for the healthfulness of the slogan or the product, there was a significant interaction effect. More significantly positive responses and increased purchase intent were elicited when the healthfulness of the slogan and the perceived healthfulness of the product were consistent. Adolescents that were highly concerned about health responded more positively towards healthy slogans. No significant differences were found between genders.
Harris et al., 2018 [40] USA	Experimental	LOW	Children (7–11 years old).	To evaluate the effects of health messages in child-directed advertising for unhealthy food products	Children’s perception of healthfulness of unhealthy products increased when associated to nutrition and/or physical activity messages. No significant differences were found between genders.
Gines Geraldo and Machado Pinto e Silva, 2012 [41] Brazil	Experimental	LOW	Children (6–10 years old)	To describe visual memory of the packaging of snacks and filled cookies in relation to nutritional status, school grade and gender	Both genders exhibited similar memory levels for colors depicted in the packaging of both products. Girls remembered imagery and characters depicted on the packaging better than boys. Nutritional status was not a significant influence on visual memory of pack features.
Anschutz et al., 2010 [42] Netherlands	Experimental	LOW	Children (8–12 years old)	To assess the effect of viewing adult-directed advertising (energy-dense, “light” products, and non-food products) on concurrent snack intake and possible moderating effects of maternal behaviors.	Food intake decreased significantly with age and increased with hunger and liking of the test food products. Children who received maternal encouragement to be thin ate more when exposed to food ads (vs. non-food ads), while children that did not receive such encouragement ate more when exposed to non-food ads. No significant main effect was found for ad types (energy-dense food, “light” food, non-food) or gender.
Castonguay and Bakir, 2018 [43] USA	Experimental	LOW	Children 5 to 11 years old	To analyze gender differences in nutritional understanding, intentions to engage in physical activity and responses to an advertisement for a unhealthy food product with and without imagery of children practicing sports	Compared to girls, boys that viewed an ad portraying physical activity were more likely to believe that eating the advertised food (sugar-coated cereal) would make them stronger, compared to girls and to boys who viewed an ad without such images.
	Descriptive, cross-sectional. Content analysis	HIGH	N/A	To analyze the content of food ads aired during children-directed TV shows.	Most ads shown during children-directed TV shows exclusively male characters, while 18.8% of ads featured female characters exclusively, and 6.7% featured males and females. Healthy food products were associated to girls, while healthy activities were predominantly associated to boys.
Childs and Maher, 2003 [44] USA	Descriptive, cross-sectional. Content analysis	HIGH	N/A	To examine the use of gender in children-directed TV food ads.	There was overrepresentation of male voices and characters portrayed in the ads, indicating that a gender bias towards male audiences exists in food advertising to children.
Skatrud-Mickelson et al., 2012 [45] USA	Descriptive, cross-sectional. Content analysis	HIGH	N/A	To estimate exposure of children to food brand impressions in top-grossing movies.	Exposure to food brand impressions varied among boys and girls depending on age and movie MPAA ratings. Girls were more exposed to brand impressions from G/PG-rated movies, while boys were more exposed from PG-13/R-rated movies.
Harrison, 2006 [46] USA	Descriptive, cross-sectional. Content analysis	HIGH	N/A	To analyze food advertising content in children-directed TV, comparing ads portraying Black and non-Black characters.	Male characters were overrepresented in both the ads that featured Black characters and those who did not.
Olivares et al.,2011 [47] Chile	Quantitative. Descriptive, cross-sectional.	MEDIUM	Children (8–13 aos)	To explore attitudes towards food ads among school children.	65% of participants expressed a liking for trying new foods and beverages promoted in TV ads. No significant differences were found between genders.
Cornwell et al., 2014 [48] USA	Quantitative. Descriptive, cross-sectional.	MEDIUM	Children (3–6 years old)	To evaluate associations between BMI and knowledge of brands of food products high in fats, salt and sugar among children	Knowledge of packaged and fast food brands was found to be a predictive factor of BMI among children. No significant differences were found by age and gender, nor according to hours spent watching TV.
Klepp et al., 2007 [49] Austria, Iceland, Portugal, Norway, Belgium, Denmark, Spain, Netherlands, Sweden	Quantitative. Descriptive, cross-sectional.	HIGH	Children (average: 11 years old)	To investigate associations between exposure to food ads in TV and reported fruit and vegetable intake among children from nine European countries.	Most children reported higher exposure to ads for unhealthy food than for fruit and vegetables, but boys reported spending slightly more time watching TV than girls. Exposure to ads for healthy foods was positively associated with reported fruit and vegetable intake.
Baldwin et al., 2018 [50] Australia	Quantitative. Descriptive, cross-sectional.	LOW	Children and adolescents (10–16 years old)	To examine associations between internet and social media behavior and unhealthy food intake	Exposure to advertising was associated with a higher intake of the advertised products. No significant differences were found between genders.
Bhawra et al.,2018 [51] Canada	Quantitative. Descriptive, cross-sectional.	MEDIUM	Adolescents and young adults (16–30 aos)	To assess support to food policies among youth in Canada.	Young women expressed stronger support than men for nutrition symbols and warnings school policies, zoning restrictions on advertising, marketing bans and maximum salt limits.
Kumar et al., 2015 [52] USA	Quantitative. Descriptive, cross-sectional.	MEDIUM	Adolescents (12–17 years old)	To analyze exposure to ads for sugar-sweetened beverages	Between 42 and 54% of the participants reported exposure to these advertisements more than once a day. Significant differences were found by age, ethnic group and parents’ educational level, as well as gender. Boys reported more frequent exposure to sugary sport beverage ads than girls.
Adachi-Mejia et al., 2011 [53] USA	Quantitative. Descriptive, cross-sectional.	MEDIUM	Children (10–13 years old)	To explore the relationship between weight status and receptivity to food advertising among adolescents.	Having a favorite ad was the chosen indicator of receptivity. Boys who reported having a favorite ad were more likely to mention an ad for beer, while girls were more likely to mention an ad for candy/sweets as their favorite.
Buijzen et al., 2008 [54] Netherlands	Quantitative. Descriptive, cross-sectional.	LOW	Children (4–12 years old)	To analyze associations between exposure to food advertising and consumption of advertised brands, advertised energy-dense food product categories and food products in general	Exposure to food advertising was significantly associated to consumption of advertised brands and energy-dense food product categories. Intra-family consumption-related communication was found to moderate the relations between advertising and food consumption. No significant differences were found by age, gender, and time spent watching TV.
Grunseit et al., 2012 [55] Australia	Quantitative. Descriptive, cross-sectional.	LOW	N/A	To examine a) opinions regarding the role of athletes in the promotion of physical activity and obesity prevention, b) attitudes towards the promotion of unhealthy food products in sports and c) health-related behaviors among young Australian athletes.	Most participants agreed that athletes should be positive role models for active lifestyles. Women tended to agree more with the proposition than men. There were also significant differences in the percentages of women that were opposed to advertising of alcohol and unhealthy food in sports and endorsement by professional athletes, compared to men.
Olivares-Cortes et al., 2017 [56] Chile	Quantitative. Descriptive, cross-sectional.	HIGH	N/A	To explore attitudes and opinions about a new front-of-pack nutrition labeling system among school-children with diverse nutritional status and socioeconomic level	Most children reported liking to be informed about the nutritional value of food products and many expressed that they would stop buying products with nutrient warning signs. There were significant differences by nutritional status and socioeconomic level, but gender was not a significant influence.
Bezbaruah and Brunt, 2012 [57] USA	Quantitative. Descriptive, cross-sectional.	LOW	Children (9–11 years old)	To determine the influence of cartoon characters in fruit and vegetable preferences	The children reported that their fruit/vegetable preferences were determined by flavor and nutritional value. There were no significant differences between genders.
Vila-López and Kuster-Boluda, 2016 [58] Spain	Quantitative. Descriptive, cross-sectional.	HIGH	Adolescents (14–17 years old)	To explore gender differences in the association between perception of food packaging cues and health motivations and weight control	Girls were more concerned about weight control and health and paid more attention to informative cues on food packaging when motivated to control weight, compared to boys. Visual cues were not relevant for both genders.
Marquis et al., 2005 [59] Canada	Quantitative. Descriptive, cross-sectional.	LOW	Children (10 years old)	To evaluate the association between eating while watching TV and food-related behaviors	Eating while watching TV was found to be significantly associated to unhealthier food choices. Significant correlations were found between the frequency of eating while watching TV, the importance given to a food’s appearance and children’s requests for advertised foods, but only among boys.
Kaur and Vohra, 2013 [60] India	Quantitative. Descriptive, cross-sectional.	MEDIUM	Children (4–11 years old)	To analyze the effectiveness of in-store food promotion strategies targeting children.	Free gifts highlighted on packaging, assortment of foods, and placement of packaged foods in shelf locations directly accessible to children were found to be among the most effective in-store promotional strategies. Food requests were more strongly affected by these influences among boys than among girls.
Vohra and Soni, 2015 [61] India	Quantitative. Descriptive, cross-sectional.	LOW	Children (4–11 years old)	To identify the variables that predict shopping behavior in retail stores among children.	Retail shopping behavior in children is influenced by food promotions in stores, the frequency with which the child accompanies their mothers to the store, the age of the parents and maternal educational level. There was no significant difference among genders.
Busse and Díaz, 2016 [62] Peru	Quali-quantitative Descriptive, cross-sectional	HIGH	Children (7–11 years old)	To explore habits regarding TV and food behaviors among Peruvian children.	Boys and girls reported different preferences in TV shows, suggesting differences in exposure to food-related TV content. Some girls demonstrated awareness of the persuasive intent in TV ads, while others did not. In contrast, all boys were aware of the persuasive intent in advertising.
Bunting et al., 2013 [63] New Zealand	Qualitative. Descriptive, cross-sectional	MEDIUM	Adolescents and young adults (16–21, 22–28 and 29–35 years old)	To evaluate perceptions and knowledge about energy drinks	Participants expressed awareness regarding the marketing of different energy drinks as predominantly “feminine” or “masculine” and the resulting manipulation of consumer choices by the food industry.
Elliott, 2009 [64] Canada	Qualitative. Descriptive, cross-sectional	MEDIUM	Children fromgrades 1 to 6	To analyze differences in attitudes and responses of boys and girls towards “fun food” marketing techniques.	Girls were more likely to choose products based on color preferences and aesthetic appeal, while boys were more interested in the interactive features of the products.

**Table 2:** Classification of included studies by theme, finding of differences by gender and methodological quality

Theme / methodological quality	Study	Methodological quality	Direction of the gender differences
Effect of food marketing on food intake among children and adolescents	Anschutz et al. 2009 [35]	Low	Boys were more influencied than girls
	Anderson et al. 2015 [32]	Low	Girls were more influencied than boys
	Keller et al. 2012 (study 2) [34]	Low	Girls were more influencied than boys
	Cornwell et al. 2014 [48]	Medium	No gender differences
	Norman et al. 2018 [31]	Medium	No gender differences
	Ueda et al. 2012 [28]	Low	No gender differences
	Buijzen et al. 2008 [54]	Low	No gender differences
	Keller et al. 2012 (study 1) [34]	Low	No gender differences
	Anschutz 2010 [42]	Low	No gender differences
	Baldwin et al. 2018 [50]	Low	No gender differences
Effect of food advertising on preferences and choice	Kaur & Vohra 2013 [60]	Medium	Boys were more influencied than girls
	Chernin 2008 [29]	Low	Boys were more influencied than girls
	Castonguay & Bakir 2018 (study 2) [43]	Low	Boys were more influencied than girls
	Marquis et al. 2005 [59]	Low	Boys were more influencied than girls
	Olivares-Cortés 2017 [56]	High	No gender differences
	Tarabashkina 2017 [30]	Medium	No gender differences
	Olivares et al. 2011 [47]	Medium	No gender differences
	Vohra & Soni 2015 [61]	Low	No gender differences
	Velazquez & Pasch 2014 [30]	Low	No gender differences
Responses to specific marketing techniques	Vila-López & Kuster-Boluda 2016 [58]	High	Girls were more influencied than boys
	Elliott 2009 [64]	Medium	Girls and boys were influencied by different attributes
	Ogle et al. 2017 [38]	Low	Boys were more influencied than girls
	Gines Geraldo & Machado Silva 2012 [41]	Low	Girls were more influencied than boys
	Hobin et al. 2012 [37]	Low	Boys were more influencied than girls
	Dixon et al. 2014 [36]	Low	Boys were more influencied than girls
	Adams & Geuens 2007 [39]	Low	No gender differences
	Bezbaruah & Brunt 2012 [57]	Low	No gender differences
	Harris et al. 2018 [40]	Low	No gender differences
Perceptions and attitudes towards food marketing and marketing restrictions	Busse & Díaz 2016 [62]	High	Girls were more vulnerable than boys
	Adachi-Mejia et al. 2011 [53]	Medium	Girls and boys were influencied by different attributes
	Bhawra et al. 2018 [51]	Medium	Girls were more likely to support policies than boys
	Grunseit et al. 2012 [55]	Low	Girls were more likely to support policies than boys
	Bunting et al. 2013 [63]	Medium	No gender differences
Exposure to food advertising and gendered marketing content	Klepp et al. 2007 [49]	High	Boys were more exposed than girls
	Childs & Maher 2003 [44]	High	Content was more directed towards boys
	Harrison 2006 [46]	High	Content was more directed towards boys
	Skatrud-Mickelson et al. 2012 [45]	High	Girls and boys were influencied by different attributes
	Kumar et al. 2015 [52]	Medium	Boys were more exposed than girls
	Castonguay & Bakir 2018 (study 1) [43]	High	Content was more directed towards boys

Table 1 presents a summary of each study’s main objective and outcomes, as well as its quality score. Table 2 illustrates the distribution of studies of low, medium and high methodological quality within each dimension of interest.

### Gender differences in the effect of food marketing on food intake among children and adolescents

A group of ten studies analyzed the differential effects of food marketing on eating behaviors among children and adolescents, particularly of exposure to food advertising in different media (TV, internet, etc.), although TV was the most frequently investigated one. Most of these studies (*n* = 8) were classified as having low methodological quality (Table 2). Only three studies [32, 34, 35] found significant differences between genders, but the gender identified as more strongly influenced by food marketing varied among studies.

An experimental study conducted in the Netherlands on children of 8 to 12 years of age by Anschutz et al. [35] reported that boys tended to eat a greater amount of food when viewing TV shows embedded with food ads vs. non-food ads, but this association was not found among girls.

Moreover, a study conducted by Anderson et al. [32] on children and adolescents (9–14 years old) in Canada found that girls with overweight/obesity were more vulnerable to marketing techniques and increased their energy intake after exposure to food ads, while girls with average weight and boys did not. Similar results were obtained by Keller et al. [34] on an investigation conducted in the USA to determine the impact of familiar fast-food branding on test-meal intake in children (7–9 years old). Girls were found to increase food intake in the presence of familiar fast-food brands, while boys ate similar amounts when presented with fast-food branded items vs. neutral food items (i.e. in plain white containers).

The seven remaining studies that analyzed the effect of exposure to food advertising did not observe significant differences between genders [28, 31, 34, 42, 48, 50, 54], although they made some noteworthy observations regarding this influence and other intervening variables. One study found that brand recognition in TV ads of products high in sugar, fat and salt was a predictor of BMI in children as young as 3 years old [48], while other studies observed that maternal encouragement to be thin [42] and intra-family communication [54] had a moderating effect in the impact of food advertising in school-aged children. Young children with overweight were found to be more responsive to branding of food items than children with average weight [34]. More recent studies have also explored the impact of online advertising. Exposure to online food ads was found to be associated with increased consumption of unhealthy food products [50] and the influence of food advertising was observed to depend on the type of ad and media [31].

### Gender differences in the effects of advertising on preferences and choice among children and adolescents

A group of nine studies explored gender differences in the effects of advertising on preferences and choice among children and adolescents, of which four [29, 43, 59, 60] generated evidence that food advertising exerts a more powerful influence on choices and preferences among male children and adolescents than among females.

An experimental study by Chernin et al. on children aged 5 to 11 years old in the USA found that exposure to food advertising increased the children’s preference for the advertised products, an effect that was more powerful among boys [29]. Also in the USA, Castonguay & Bakir [43] evaluated attitudes towards maintaining an active lifestyle among school-aged children after exposure to advertising portraying physical activities versus neutral ones. Boys manifested a greater intention to engage in physical activity when exposed to advertising depicting sports, compared to girls overall and to boys who only viewed neutral ones. The authors concluded that boys are more likely to believe that the energy-dense, low quality foods depicted in these advertising will help boost their physical performance than girls.

In Canada, Marquis et al. [59] evaluated how eating while watching TV affected purchase requests among school-aged children. Boys reported asking their parents to purchase the advertised products more frequently than girls, which suggests that boys were more vulnerable to food advertising than girls. Similar results were obtained by Kaur and Vohra [60] in a cross-sectional study regarding the influence of promotional marketing techniques in retail stores among school-aged children in India. The analysis of questionnaires administered to the children’s mothers showed that these promotion strategies affected boys’ purchase requests more strongly than girls’.

The remaining five studies that assessed the impact of advertising on preferences and choice did not show significant differences between genders [30, 33, 47, 56, 61]. However, these studies shed light on the specific ways in which this influence operates. These studies did not only confirm advertising and in-store promotional strategies to be powerful influences on food choice and preferences among children and adolescents [61], but in some cases they also identified other factors that moderate this effect, such as nutritional knowledge [30], the degree of attention to advertisements [33] and socioeconomic variables other than gender [56, 61].

### Gender differences in responses to specific marketing techniques among children and adolescents

Six [36–38, 41, 58, 64] of the nine studies included in this category found that male and female children and adolescents responded differently to specific marketing techniques, such as promotions, cartoon characters or sport celebrities in food packaging, and offer of toys as premium. An experimental study conducted in the USA by Ogle et al. [38] focused on the influence of depictions of licensed cartoon characters in food packaging on the attention and food preferences of school-aged children, finding that children prefer products depicting characters of their same gender. In Australia, Dixon et al. [36] also conducted an experimental study in a similar age group, where male children were observed to be more attracted to products that showed sport celebrities or that included toys, compared to girls. In an experimental study on visual memory among school children in Brazil, Gines Geraldo and Machado Pinto e Silva [41] found that girls could remember images and characters to a greater degree than boys, although no significant differences were found between genders in the capacity to remember colors.

Two studies focused on marketing techniques that involve active interaction with products. Using an experimental design, Hobin et al. [37] found that Canadian children were more likely to choose healthier products if toys were offered as a premium only with healthier foods, but this effect was stronger in boys than in girls. In turn, by means of focal groups, Elliot [64] explored the marketing techniques involved in “fun foods” and observed that products with enhanced interactive features were more appealing to male school-children, whereas female choices were more determined, on the one hand, by aesthetic values, such as package attractiveness, color and overall design and. On the other hand, they were determined by associative values such as those evoked by the food or package relating the food to, for example, a movie, a family outing, a feeling, among others.

As for adolescents, Vila-López and Kuster-Boluda [58] explored the importance of visual and information cues in food packages in the attitudes and preferences of adolescents in Spain. While visual cues were not found to be a significant influence for either gender, information cues were observed to affect female adolescents more strongly than males, because females tended to be more worried about weight control and health overall and these factors played more heavily on their food choices.

Of three studies that did not observe significant differences between genders, one found that the interaction of healthy/unhealthy slogans with healthy/unhealthy products had a significant influence in purchase intent among 15-year-olds in Belgium [39]. Another study assessed the potential of using cartoon characters to promote fruit and vegetable consumption among children, with varying results by type of character and age of the audience [57]. The third paper corroborated the power of health claims and their impact on children’s perceptions of relative healthfulness of food products with low nutritional quality, without finding any difference between genders [40].

### Gender differences in perceptions and attitudes towards food marketing and marketing regulation initiatives

This group includes five studies [51, 53, 55, 62, 63] whose findings suggest that children and adolescents presented some level of awareness regarding the persuasive intent behind marketing, particularly gendered advertisements. The studies also show that males and females have different perspectives regarding the need for regulation of food marketing.

Focus groups conducted by Bunting et al. [63] in New Zealand explored perceptions involving the marketing of energy drinks among adolescents and young adults (16 to 35 years old). All participants demonstrated being highly aware of the fact that energy drinks advertising targeted preferentially men or women, and that this implies a certain level of manipulation of audiences to choose products based on gendered perceptions. In contrast, a similar design implemented by Busse and Díaz [62] among school-aged Peruvian children found that all boys were aware of the persuasive intent in TV advertising, but some girls did not, suggesting a greater vulnerability to advertising persuasion in girls than in boys.

Adachi-Mejia et al. [53] assessed receptivity to TV advertising among 10 to 13 years old in the USA, in which having a favorite advertisement was used as a measure of receptivity. Although both girls and boys showed similar receptivity levels, among respondents who declared having a food ad as their favorite, boys were more likely to prefer a beer ad than girls. Conversely, girls were more likely to name a candy ad than boys.

Bhawra et al. [51] and Grunseit et al. [55] gauged the support for food marketing regulations among adolescents and young adults (16 to 30 years old) in Canada and young Australian athletes (15 to 23 years old), respectively. In both cases, women were more likely to support food marketing restrictions and related policies. Additionally, female athletes also showed a greater level of disagreement with the promotion of unhealthy foods associated to sports than men [55].

### Gender differences in exposure to food advertising and gendered marketing content targeting children and adolescents

There were six studies [43–46, 49, 52] that explored either gender differences in exposure intensity or gendered marketing content. Results suggest that boys were exposed to food advertising more frequently than girls and that there is a gender bias in food marketing.

When administered questionnaires, boys reported that they viewed/listened to food advertising more often and spent more time watching TV than girls, both in the USA [52] and Europe [49].

Studies focusing on advertising content found significant differences in both the frequency with which each gender was portrayed and the associated types of messages or foods. Castonguay and Bakir [43] observed that only 18.8% of TV advertisements in the USA had exclusively female characters and 6.7% showed both genders, while 74.6% showed only males. Moreover, there was an association between food healthfulness and gender, where women were more likely to be associated to healthful products and men to unhealthful ones. In the UK, Childs and Maher [44] also found that TV advertisements were gender bias towards boys, who were portrayed more often, either in image or as voice-over, than girls. Harrison [46] also included ethnicity in their study of gendered content in TV advertising, dividing advertisements into two groups: those portraying Black characters and those that did not. They observed that male characters were overrepresented in both groups (62.7 and 65.4%).

Skatrud-Mickelson et al. [45] followed a very different approach to this issue and estimated exposure by combining audience composition data, ticket sales for the top-20 box-office movies in the USA, and the number of times specific food brands appeared on screen in each of these movies. Their results suggest that gender differences in exposure to advertising were determined by the interaction of age and movie age rating. Exposure among girls was greater than boys for ages 6 to 11 and 12 to 17 in G/PG rated movies, while boys of all ages had a higher exposure in PG-13 and R-rated movies.

## Discussion

Little is known about the role of gender in food marketing to children and adolescents. This narrative review revealed that gender may play an important role in the development of food marketing techniques and how children respond to them. Literature [16] shows how the election of the foods and different food behaviors may be related to social expectations and gender stereotypes. Social media may contribute to the development of gender stereotypes in children and adolescents by reinforcing gender roles [65].

However, the results found are not consistent. For example, with regards to the effect of exposure to food marketing on food intake, three of the studies included in this study [32, 34, 35] find differences between boys and girls. Whereas in two studies [32, 34] girls seem to be the most influenced by marketing, the third one differs and concludes that boys seem to be more affected [35]. These studies do not inquire into the reasons for these differences and, if they do, these are stated in a superficial way.

In the case of the studies that analyze the exposure to food marketing and the gendered advertising content, five [43, 44, 46, 49, 52] of the six studies included in this dimension, found that the advertising content was mostly directed to boys. Likewise, all the studies that analyze the effects of the advertisements in the elections and preferences found that advertisements have more influence on boys. Nevertheless, only some of the studies [43, 44, 52, 56] analyze the possible causes for this difference.

The masculine predominance in advertising contents could be explained as a cultural bias [44]. Food advertising reinforces, in this way, gender stereotypes built socially. Gender stereotypes determine the practices in which boys and girls are represented in advertisements. For example, the fact that boys appear more frequently in advertisements that represent a physical activity compared to girls reinforces the idea that sports are not for women [43]. In addition, the association between the marketing of certain products with the male strength [43] could make boys feel more persuaded to buy this kind of product. It is recognized that there is the need of a more profound analysis on socially built stereotypes [29], and how this can be embodied in advertisements with a consequent impact on the food preferences of girls and boys.

Regarding the different marketing techniques implemented, this revision found that, although food products do not have a gender per se, marketing strategies are constructed in such a way that products have become gendered, resulting in foods that are more appealing to girls or to boys depending on the promotional techniques applied [36–38, 41, 58, 64].

Another possible explanation for gender differences mentioned in the studies [52] refers to the existence of a differential consumption between boys and girls [56]. In this case, it would be interesting to explore with further detail the motives that explain the difference in the consumption of food products between boys and girls.

The studies also bring to light the many variables that could mediate the association among advertising, gender, and eating behaviors, such as weight status, nutritional knowledge, advertising techniques, type of media, brand awareness, intra-family pressures, and others. It is important to understand the way in which the variables that mediate the correlation between publicity exposition and eating behaviors are affected by gender difference. In this respect, the existing literature is still scarce [16]. In addition, it is important to highlight that, the studies that do mark a difference between genders, the origin of this difference is not well problematized. The inclusion of additional theoretical frameworks used in the analysis of gender and media, such as social cognitive theories and stereotype threats would be relevant to analyze the results and expand the depth of the research results [65].

### Strengths and weaknesses of the selected studies

The main strength of this review is that it contributes to the systematization of data regarding the role of gender in food advertising and in providing new research lines for future studies.

One limitation of this study is that the body of research reviewed is heavily biased towards TV advertising, with very few studies focusing on the impact of relatively new marketing channels, such as online games or social media platforms. Another limitation is the lack of common definitions for key variables, such as “persuasion” or “attention”. It is difficult to compare results, as there is no standardization in the measurement of outcome variables. It must be taken into consideration that *n* = 3 of the investigations incorporated by this study [51, 55, 63]) include young adults (over 18 years) within their analysis. Therefore, the results of these studies may not represent our universe of study exclusively.

The methodological quality varies among the studies, although certain dimensions seem to have been explored using consistently more sound methodologies than others, such as gender differences in exposure to advertising and gender bias in advertising content. In contrast, most of the studies assessing the effect of marketing on food intake were of low quality. However, no associations between study quality and the identification of gender differences in any of the issues of interest for this review were identified.

Perhaps the most serious shortcoming of the work discussed in this review is the poor development of a common theoretical framework to investigate gender issues. Most of the studies reviewed here have taken a simplistic approach that limits the question to biological, genetic or metabolic differences between the sexes, which also precludes any possible consideration of other, non-binary gender identity. In this way, the necessity of developing approaches that are able to differentiate sex from gender has been recently stated. This is necessary so as to fill a vacancy that exists in the literature respect transgender and gender nonconforming population (TGNC) and, in this way, achieve the inclusion of these populations in the development of investigations and nutrition programs [66]. In addition, in order to make a meaningful contribution to gender studies, the research of the influence of food marketing on the eating behaviors of girls and boys must account for the cultural, economic and social factors that come into play in the construction of gender identities and how these factors influence the relationship between food advertising and people, mediated by social expectations and stereotyped “masculine” and “feminine” behaviors, attitudes, and preferences [67].

Finally, most of the studies were conducted in Europe, the USA or Australia, with little to no representation of the developing world.

### Areas identified for future research

The body of work explored in this scoping review is a testimony to the increasing interest of the academic community in exploring gender-related determinants to eating behaviors and its implications for disparities in public health. We have identified issues that would particularly benefit from further inquiry, especially in the developing world, including gender stereotypes portrayed and reinforced in food advertising and how they have changed over time, how exposure to advertising in children and adults varies in relation to their gender, the impact of new media and the role of influencers. The majority of studies focused on the gender difference among children and adolescents concerning the effect of food marketing on food intake and the effects of advertising on preferences and choice showed no differences based on gender. These results can be an effect of study limitations and require further investigation.

## Conclusions

This scoping review revealed that gender may play an important role in the development of food marketing techniques and how children respond to them. In addition, the review highlights the necessity of continuing developing studies that not only evidence the difference between sex but of studying the difference from a gender perspective.

Gender is a transversal dimension that interacts and enhances all other forms of health disparities, and it must be considered in policies that address obesogenic environments. It is necessary to develop more studies that do not only show the differences, but that understand the sources of the differences and that exceed the definitions of gender based only on biological aspects. These investigations will be of great use for the development of transversal gender policies.

## Data Availability

Not applicable.
